# Long-term aquarium records delineate the synchronized spawning strategy of *Acropora* corals

**DOI:** 10.1098/rsos.240183

**Published:** 2024-05-29

**Authors:** Yusuke Sakai, Hiromi H. Yamamoto, Shinichiro Maruyama

**Affiliations:** ^1^ Department of Biology, Graduate School of Science, Osaka Metropolitan University, Osaka 558-8585, Japan; ^2^ Okinawa Churashima Foundation Research Institute, Okinawa 905-0206, Japan; ^3^ Department of Integrated Biosciences, Graduate School of Frontier Sciences, The University of Tokyo, Kashiwa, Chiba 277-8562, Japan

**Keywords:** coral, reproduction, aquarium, synchronous spawning, statistical analysis

## Abstract

Aligning spawning timing with seasonal environmental changes is critical for both terrestrial and aquatic organisms. However, mechanisms to regulate reproductive activity in response to environmental factors are not well understood, partly owing to the technical difficulty in maintaining detailed long-term observational data of the reproductive activities in the same population across years. In this study, we present an application of the aquarium system to examine the long-term spawning properties of corals. Spawning records over a 15-year period at the Okinawa Churaumi Aquarium revealed that the spawning timing of *Acropora* corals in aquarium tanks aligned well with that of wild corals from a neighbouring reef. Using the aquarium population as a model, we investigated the relationship between key environmental factors and the timing of the first and peak spawning dates of *Acropora* corals during a 15-year period. The results suggest that the spawning window of each spawning season is largely influenced by the water temperature and that the timing of peak spawning can be fine-tuned in response to environmental fluctuations. This behavioural feature can prevent synchronous spawning events during unfavourable environmental conditions and increase long-term reproductive reliability.

## 1. Introduction

The timing of biological events is largely determined by seasonal fluctuations in environmental factors. Scleractinian corals follow an annual gametogenesis cycle and exhibit high spawning synchrony within and among species [1,2]. Predicting coral spawning is crucial for understanding coral population dynamics, community ecology and the conservation of coral reef ecosystems, which have faced significant decline in recent years. However, despite its importance, the precise mechanisms governing the fine-tuning and coordination of coral spawning timing remain elusive.

There are multiple time scales for the regulation of synchronous spawning in corals: spawning month (seasonal synchrony), spawning day (lunar synchrony) and spawning hour (diel synchrony) [3]. Among them, the prediction of spawning day is particularly difficult owing to inter-annual, inter-regional and inter-species variations [4]. It has been documented that *Acropora* corals are sensitive to various environmental factors, especially seawater temperature [5–8], which is suggested to contribute to the variation in spawning day. Conversely, the spawning day of some merulinid coral species precisely follows the lunar cycle, with spawning occurring within a narrower time window [8]. Lin *et al*. [9] reported that the spawning day of the coral *Dipsastraea speciosa* is determined by the presence of a dark period from sunset to moonrise and, as such, the spawning period is limited to specific lunar days (5–8 days after full moon) [9].

Detailed observation and, in some cases, manipulation experiments in laboratory settings are useful to disentangle and reductively analyse the complex interaction between environmental factors and spawning days. However, maintaining continuous spawning experiments over several years in a laboratory setting is technically challenging. In this study, we propose that long-standing records of coral spawning in public aquariums can provide an important link between laboratory and field environments. We obtained data covering the entire spawning period for the 15 years from 2003 to 2017 from Okinawa Churaumi Aquarium. From these data, we analysed in detail the environmental factors that may have influenced the inter-annual variation in spawning days, leading to a plausible strategy for explaining the spawning behaviour of *Acropora* corals.

## 2. Material and methods

### 2.1. Spawning observation of *Acropora* sp. in open-air tanks at Okinawa Churaumi Aquarium

**Figure 1.**
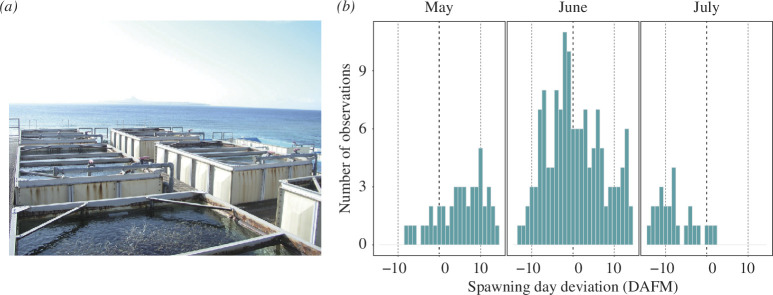
(***a***) The open-air breeding tanks in Okinawa Churaumi Aquarium. (***b***) Pattern of spawning timing in *Acropora*. Histogram shows spawning day deviation from the full moon day (Day 0, Bold dashed line) in each month from May to July. The vertical axis indicates the number of tanks in which spawning was observed. Data for all 15 years were combined.

Spawning observations were conducted in open-air flow-through tanks at Okinawa Churaumi Aquarium (26°41′40.1″ N, 127°52′41.28″ E) for 15 years (2003–2017) (figure 1*a*) [10]. The *Acropora* colonies were kept in approximately 20 outdoor tanks (500–60 000 l) fitted with an open-water flow-through system, located on the coast adjacent to the natural reef of the colonies. Seawater was drawn in continuously, without filtering, from a site located 200 m offshore at a depth of 20 m and the seawater was exchanged once per hour. In these tanks, a total of approximately 3000 branching *Acropora* colonies were maintained throughout the observation period. The majority consisted of *A. intermedia* colonies, with a small number of *A. microphthalma* and *A. tenuis* colonies, approximately one-tenth of *A. intermedia* colonies. Breeding tanks were monitored from 6.30 to 23.00 daily from the high tide in May to the high tide in July each year, and the presence/absence and scale of spawning were recorded. We assigned spawning scale values for each tank for each monitoring day by visual check: a spawning scale value of 3 when greater than or equal to 75% of corals in the tank spawned, 2 (approximately 50%), 1 (less than or equal to 25% or no spawning scale information available), or 0 (no spawning). Spawning records of wild populations of *A. intermedia*, *A. microphthalma* and *A. tenuis* (the same species as in the aquarium population) from reef sites in the Northern Hemisphere from 2006 to 2017 were surveyed and four reefs at different distances from Okinawa Churaumi Aquarium (26°38′24.0″ N, 127°52′12.0″ E), in which records were available for 5 years or more, were selected [4]. The approximate distances from the aquarium are 8 km to Sesoko, 81 km to Akajima, 787 km to Lyudao (Taiwan) and 1413 km to Cabarruyan Island (Philippines). Spawning coincidence was defined as a ratio of the number of days in which spawning was observed in both aquarium and field to the one in which spawning was observed in the field.

### 2.2. Environmental variables

Five environmental variables were selected in this study: water temperature, the difference in water temperature between consecutive days (∆WT), precipitation, wind speed and solar flux. Water temperature at the surface of each open-air tank was measured three times per day at set hours (9.00, 13.00 and 16.00) using a digital water thermometer. For statistical analyses, we calculated the daily mean values of water temperature from two specific tanks (called ‘M4’ and ‘S1’) at the time points 9.00 and 16.00. Daily solar flux was derived from the Adjusted All-Sky Surface Spectral Shortwave Down Flux values from NASA’s Clouds and the Earth’s Radiant Energy System Synoptic (CERES-SYN1 deg) Edition 4. A single value for each day was determined by averaging the values from the region bounded by 26°30′00.0″ N to 27°30′00.0″ N and 127°30′00.0" E to 128°30′00.0″ E, at 1° × 1° spatial resolution. Daily precipitation amount (mm) and mean wind speed (m s^−1^) were obtained from the Nago Local Meteorological Observatory (Japan Meteorological Agency, https://www.jma.go.jp/jma/indexe.html), located approximately 14 km southeast from the Okinawa Churaumi Aquarium. The mean values of each environmental variable were calculated and standardized to Z-score values (a mean of 0 and a s.d. of 1) for four time ranges in accordance with a previous study [7]: (1) −1 to −30, (2) −31 to −60, (3) −61 to −90, and (4) −91 to −120 days after the full moon (DAFM) of the month of spawning for each year.

### 2.3. Statistical analysis

The sum of the spawning scale values (0–3) from all tanks observed for each day was defined as a ‘daily spawning score’ to calculate the following variables for spawning activities. Three spawning variables were used for statistical analysis in this study: the first spawning date (the date at which spawning was observed for the first time in each year), peak spawning date (the date at which the highest daily spawning score was recorded in each year) and total number of spawning days for each year. Multiple linear regression models were constructed to analyse the effects of the five environmental factors selected (explanatory variables) on each of the three spawning variables (response variables). A full regression model was constructed for each of the four time ranges, grouping together the data across all 15 years. In addition to the full models, we analysed the best-fit models for each time range. Models were selected based on Akaike information criteria (AIC) values. All analyses were performed using R, version 4.2.1 (https://www.r-project.org/).

## 3. Results

### 3.1. Spawning timing in the aquarium and wild coral populations

**Figure 2.**
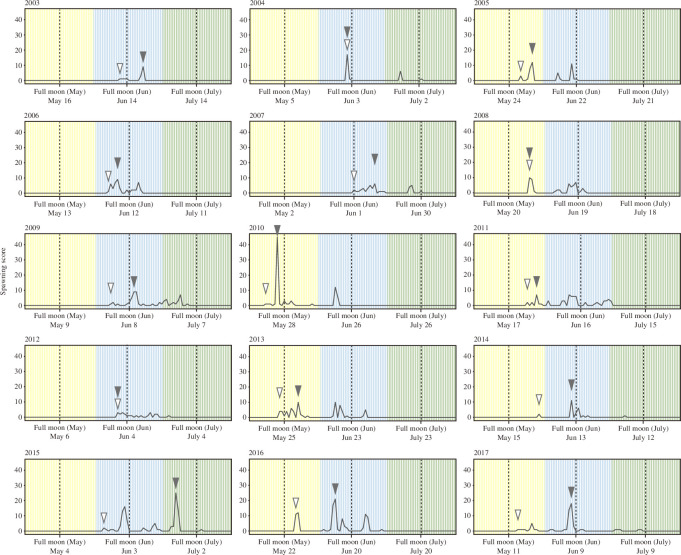
Observational records of daily spawning scores, for May (yellow), June (blue) and July (green) across all 15 years. Daily spawning scores are the combined total spawning scales (0–3) across all tanks for each day (see §2 for details). Open and solid arrowheads indicate the first and peak spawning dates of each year, respectively. The dashed line indicates the day of full moon in each month.

**Figure 3.**
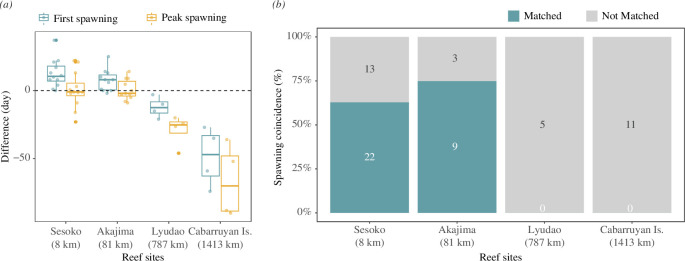
(***a***) Comparisons of spawning timing between the aquarium and field populations. The differences indicate how many days later the field records of the first spawning dates of wild populations are compared to the first (cyan) or peak (yellow) spawning dates in the aquarium population. The distances from each reef site to Okinawa Churaumi Aquarium are shown in parentheses. (***b***) Spawning coincidence of six reef sites. The numbers of field-recorded spawning events ‘matched’ and ‘not matched’ to the aquarium records are shown.

*Acropora* spawning in the Okinawa Churaumi Aquarium took place from May to July, mainly around the June full moon, for 15 years from 2003 to 2017 (figure 1*b*). The spawning pattern in the aquarium population varied among years (figure 2), with several years showing two or more discrete spawning peaks over two consecutive months (i.e. 2004, 2010 and 2015), while other years showed no clear peaks (i.e. 2007, 2011 and 2012). We compared the spawning dates in the aquarium corals with those from the field records of three *Acropora* corals (*A. intermetia*, *A. microphthalma* and *A. tenuis*) which are the same species as the aquarium population. Accordingly, the aquarium population showed higher synchronous spawning with wild corals in the neighbouring reefs, Sesoko and Akajima, than those in the distant reefs, Lyudao and Cabarruyan Island (figure 3*a*). Moreover, the spawning coincidence values were high with the two neighbouring reefs, but less so with the two distant reefs (figure 3*b*). Especially, the peak spawning dates of the aquarium population were matched well with the recorded spawning timing of the wild corals from the neighbouring reefs (figure 3*a*; electronic supplementary material, figure S1). These results support the notion that the aquarium population can serve as a good representative for investigating the spawning profiles of the local wild population.

### 3.2. Environmental factors affecting spawning timing in the aquarium corals

We then statistically analysed the correlation between environmental factors (daily means and day-to-day differences of water temperature, solar flux, wind speed and precipitation) on the spawning timing of the aquarium corals. Table 1 shows the results of regression analyses on the spawning day deviation from full moon against the five environmental variables. The mean water temperature in time range (1), particularly in time range (2), as well as the solar flux in time range (3), exhibited a strong negative correlation with the first spawning date. A similar trend was seen for the peak spawning date, with the mean water temperature for time ranges (1) and (2) being negatively correlated with the peak spawning date. This indicates that high water temperature accelerated the spawning process within the lunar cycle-dependent spawning period. Additionally, daily precipitation and solar flux were negatively correlated with the peak spawning date. The total number of spawning days in each year, considered as an index for the range of possible spawning days each year, showed a positive correlation with the water temperature in time range (3), and a negative correlation with precipitation in time range (4) (electronic supplementary material, table S1). A summary of the results for the best-fit models is shown in electronic supplementary material, table S2.

**Table 1.** Effect of environmental factors on the deviation from full moon of the first and peak spawning dates. Results of linear regressions of DAFM values for the first and peak spawning date across 11 years are shown. Individual regression models for each time range were constructed, mean values of five environmental factors for each time range were set as explanatory variables. The peak spawning dates for each year were determined from the spawning scores (figure 2). See §2 for details of statistical analysis. CI: confidential intervals. Asterisks indicate significant differences.

time range	variables	first spawning date				peak spawning date			
(1)	fixed effects	β_coefficent_	lower 95% CI	upper 95% CI	*p* value	β_coefficent_	lower 95% CI	upper 95% CI	*p* value
	(intercept)	0.154	−2.24	2.55	0.903	1.23	0.216	2.25	0.0490*
	water temperature	−4.46	−7.89	−1.04	0.0378*	−3.94	−5.70	−2.18	0.00320**
	ΔWT	−0.529	−3.29	2.23	0.718	−0.869	−2.38	0.640	0.296
	precipitation	−4.16	−8.39	0.0733	0.0955	−5.15	−7.03	−3.28	0.00102**
	wind speed	3.09	−0.0433	6.23	0.0945	−0.857	−2.33	0.622	0.293
	solar flux	−2.11	−7.00	2.77	0.425	−3.62	−5.74	−1.50	0.0123*
(2)	fixed effects	β_coefficent_	lower 95% CI	upper 95% CI	*p* value	β_coefficent_	lower 95% CI	upper 95% CI	*p* value
	(intercept)	0.154	−2.95	3.25	0.925	1.23	−0.614	3.08	0.232
	water temperature	−7.69	−12.0	−3.40	0.00983**	−4.89	−8.11	−1.67	0.0206*
	ΔWT	1.17	−2.89	5.22	0.591	−0.414	−2.89	2.06	0.752
	precipitation	1.31	−2.91	5.52	0.607	−2.41	−5.54	0.725	0.176
	wind speed	−2.10	−5.86	1.66	0.311	−2.08	−4.33	0.168	0.113
	solar flux	0.110	−3.64	3.86	0.956	−1.79	−5.02	1.44	0.314
(3)	fixed effect	β_coefficent_	lower 95% CI	upper 95% CI	*p* value	β_coefficent_	lower 95% CI	upper 95% CI	*p* value
	(intercept)	0.154	−3.22	3.53	0.931	1.23	−2.45	4.92	0.534
	water temperature	−3.35	−7.49	0.788	0.157	−2.40	−7.31	2.51	0.369
	ΔWT	0.437	−3.70	4.57	0.842	1.40	−4.64	5.73	0.842
	precipitation	−0.414	−4.22	3.40	0.838	−1.40	−5.42	2.62	0.517
	wind speed	−2.67	−6.85	1.50	0.250	−1.60	−7.27	4.06	0.597
	solar flux	−5.49	−9.65	−1.33	0.0361*	−3.75	−8.02	0.530	0.130
(4)	fixed effect	β_coefficent_	lower 95% CI	upper 95% CI	*p* value	β_coefficent_	lower 95% CI	upper 95% CI	*p* value
	(intercept)	0.154	−3.91	4.22	0.943	1.23	−1.47	3.94	0.402
	water temperature	0.0514	−5.11	5.21	0.985	0.606	−3.71	4.92	0.791
	ΔWT	−2.64	−9.44	4.15	0.471	−1.47	−6.35	3.42	0.575
	precipitation	0.0501	−4.77	4.87	0.984	−4.21	−7.93	−0.480	0.0625
	wind speed	−2.00	−7.18	3.17	0.473	0.189	−3.48	3.86	0.922
	solar flux	−3.38	−10.2	3.47	0.365	−5.08	−10.0	−0.129	0.0842

## 4. Discussion

In this study, records of *Acropora* sp. coral spawning in semi-outdoor aquaria at the Okinawa Churaumi Aquarium enabled a detailed analysis of the spawning behaviour of the same coral population over the entire course of a single spawning period, across multiple spawning seasons. The key advantage of this dataset was the reliability and precision with which the first spawning dates, and particularly, the peak spawning dates for each year could be recorded; two dates that are difficult to systematically characterize in the field. Given this accuracy, the relationship between environmental factors and spawning behaviour over the later stages of gametogenesis could be investigated.

Our analysis shows that both the first spawning date and peak spawning date are significantly affected by the water temperature during the 60 days immediately preceding spawning (i.e. time ranges (1) and (2)) (table 1), while no significant correlations between the total number of spawning days and environmental factors were detected in these time ranges (electronic supplementary material, table S1). Importantly, the timing of the peak spawning date is also influenced by the levels of precipitation and solar flux in the 30 days immediately preceding spawning (i.e. time range (1)), with peak spawning dates tending to be earlier following higher levels of precipitation or solar flux. These results suggest that the spawning window (the duration of spawning following the first spawning date for each year) is largely determined by the water temperature, whereas the timing for the synchronous spawning within that window (i.e. peak spawning) may be fine-tuned in response to multiple environmental factors, including water temperature, in the time period immediately preceding spawning. For instance, precipitation and solar flux influenced the peak spawning date but not the first spawning date, this may be owing to both rainwater, heated up in the atmosphere in early summer, and solar radiation, transiently increasing the water temperature in the late stages of gametogenesis.

Assuming that *Acropora* determines the spawning window based on water temperature and that it is advantageous for *Acropora* to spawn simultaneously with as many other individuals as possible, how can we explain the observed trends in spawning timing in *Acropora*? Some subpopulations may begin spawning when they first detect the appropriate water temperature (i.e. the first spawning date). Other subpopulations may stand by, waiting until other environmental factors (e.g. rainfall and solar radiation) meet the desired criteria within the set spawning window, thereby fine-tuning spawning timing. This strategy reduces the risk of passing the spawning window without spawning at all while increasing the chance of synchronous spawning during more favourable environmental conditions. Recent studies suggest that split spawning (synchronous spawning events in two consecutive months) may benefit *Acropora* by realigning spawning timings with seasonal environmental signals [11] and increasing the reliability of larval supply [12]. These spawning phenotypes may also be consistent with an evolutionary strategy called the bet-hedging, which decreases temporal fitness variation by, for example, plastically diversifying individual phenotypes to cope with unpredictably varying environmental conditions [13–16], allowing corals to diversify their spawning timing within an optimal environmental window.

This study highlights the limitations and benefits of semi-outdoor aquaria as a model system for coral research. It provides a means to study coral behaviour in a more controlled environment than in the field (but less than in laboratories). Moreover, it offers an opportunity to mimic natural conditions more than other laboratory systems (but less than in the field). The aquarium system allowed for detailed tracking of the spawning behaviour of almost all individuals in each tank throughout the entire spawning window. It also facilitated the delineation of the distinct effects of environmental factors on the timing of both the first and peak spawning dates. Achieving such outcomes would have been very challenging from either field or laboratory experiments. In fact, the field observations showed that the spawning timing of the local wild population near the aquarium was well matched to the peak spawning dates of the aquarium populations (figure 3*a*, electronic supplementary material S1), suggesting that, partly owing to the technical difficulties of monitoring spawning in the field throughout the entire window, some of the first spawning dates previously reported in the field may be more equivalent to the peak spawning dates being reported here. Using the aquarium system, future studies could aim to identify environmental factors that trigger synchronous spawning by manipulating physical and biological factors to examine the effects on peak spawning dates. The limitations of this study include that our data were collected under conditions unique to the aquarium, such as limited water volume and less genetic diversity, which would substantially differ from true reef conditions. However, with almost no tidal action that has been considered an important factor for synchronous spawning [2,3], this system effectively screened out the tidal effects from other factors regulating the synchrony. Future research should examine the effect of these conditions on spawning behaviour in detail. This will provide important clues for assessing the variability of spawning behaviour in the field.

## Data Availability

All the data are available online from Zenodo [17]. Electronic supplementary material is available online [18].
